# Individual strategies in the rat gambling task are related to voluntary alcohol intake, but not sexual behavior, and can be modulated by naltrexone

**DOI:** 10.3389/fpsyt.2022.931241

**Published:** 2022-12-08

**Authors:** Nikita Tjernström, Erika Roman

**Affiliations:** ^1^Neuropharmacology and Addiction, Department of Pharmaceutical Biosciences, Uppsala University, Uppsala, Sweden; ^2^Department of Anatomy, Physiology and Biochemistry, Swedish University of Agricultural Sciences, Uppsala, Sweden

**Keywords:** copulatory test, ethanol, opioid antagonist, impulsivity, gambling disorder (GD)

## Abstract

**Introduction:**

Gambling disorder (GD) is the first non-substance or behavioral addiction to be included in substance-related and addictive disorders in DSM-5. Since GD is a younger phenomenon relative to alcohol and substance use disorders, little is known about potential unique features in GD and to what extent characteristics are shared with alcohol and substance use disorders. The rat gambling task (rGT) is used to study decision-making in rats. This study aimed to identify individual differences in rGT strategies and explore the stability of these strategies over time. Moreover, motor impulsivity, sexual behavior, and voluntary alcohol intake were examined in rats with different rGT strategies. Finally, the response to naltrexone on performance in rats with different rGT strategies was investigated.

**Methods:**

Male Lister hooded rats (*n* = 40) underwent repeated testing in the rGT, repeated copulatory behavioral tests, and 7 weeks of voluntary alcohol intake through a modified intermittent two-bottle free-choice paradigm. Finally, rats were treated with naltrexone prior to testing in the rGT.

**Results:**

The results revealed individual choice strategies in the rGT that were stable over time, even after multiple interruptions and other behavioral testing. The rats with a risky choice strategy displayed higher motor impulsivity and voluntary alcohol intake than the other groups. No difference in sexual behavior was found between the different rGT groups. Finally, in all rats irrespectively of rGT strategy, treatment with naltrexone decreased the number of completed trials and premature responses, and increased omissions, which indicates an overall lowered motivation.

**Discussion:**

In conclusion, rats with risky rGT strategies had higher voluntary alcohol intake but not elevated sexual behavior, indicating shared underlying mechanisms between rGT strategies and alcohol intake but not natural rewards in terms of sexual behavior. Finally, naltrexone treatment resulted in an overall lowered motivation in the rGT.

## Introduction

Gambling disorder (GD) is the first non-substance or behavioral addiction to be included in substance-related and addictive disorders in the Diagnostic and Statistical Manual of Mental Disorders, 5th Edition (DSM-5) (1). The inclusion in this category was due to the mounting evidence that GD shares many features with alcohol and substance use disorders (2, 3), including altered processing in brain reward networks (4). However, with GD being a younger phenomenon relative to alcohol and substance use disorders, there is still more to discern about potential unique features in GD and to what extent characteristics are shared with alcohol and substance use disorders, as well as natural rewards.

One of these shared features is reward-related decision-making, which is known to be an important feature of addictions (5) and is also affected by GD (6). The Iowa gambling task (IGT) is frequently used to study impaired reward-related decision-making in humans. The IGT contains options associated with frequent small rewards and small losses as well as options associated with large rewards and large losses. To achieve the optimal decision-making strategy, the participants need to prefer the options with small rewards that are less attractive in the short term but, due to the small losses, are more advantageous in the long run (7). Individuals with GD display deficits in decision-making when performing the IGT (8, 9), and poor performance on the IGT is predictive of problem gambling (10). The rat gambling task (rGT) is based, in part, on the IGT and enables studies of decision-making in rats (11). Notably, performance in the rGT shares features with that of humans in the IGT (12). We and others have shown that the majority of rats learn and maintain a stable choice on the most advantageous option in the rGT (11, 13–16). However, large individual differences exist, and a subset of rats prefer the safest and less advantageous option, while a different portion of rats prefer the most disadvantageous and riskiest option (15). How stable such individual rGT strategies are over time remains to be investigated.

Impulsivity has been linked to alcohol use disorders (AUDs) in humans and to alcohol intake in animal studies (17). Similarly, deficits in impulsivity have also been associated with GD (18–20). Impulsivity can be divided into motor disinhibition (impulsive action) and impulsive decision-making (impulsive choice) (21). In the rGT, the preference for choices associated with larger immediate gains but greater overall net losses reflect aspects of choice impulsivity (22). Using an inter-trial interval (ITI) extension, in which the animals need to withhold from making a response for a longer duration than normal, aspects of motor impulsivity can be investigated (13). In previous studies using the rGT, no associations between motor impulsivity and decision-making were revealed (11, 23, 24). However, a meta-analysis of 13 experimental cohorts demonstrated a negative correlation between advantageous choices and motor impulsivity (13). In the present study, an ITI extension was used to assess motor impulsivity in rats with different gambling strategies.

GD and AUDs are comorbid; problem gamblers are 3.3 times more likely to have AUDs (25), and an epidemiological survey showed that 73% of patients with GD also had AUDs (26). A meta-analysis revealed that decision-making deficits in the IGT were associated with both AUDs and GD but more pronounced in GD than in AUDs (27). Blood alcohol levels have been negatively associated with performance in the IGT (28), and binge-drinking individuals made less advantageous choices (29, 30). The effects of alcohol exposure on behavior in a modified rGT revealed that acute exposure had small effects on choice behavior, while repeated alcohol exposure increased risky choices (31). Conversely, a follow-up study, which first divided rats into low- and high-alcohol drinkers, found that high drinkers performed better on the rGT and acute alcohol treatment increased optimal decision-making (32). In the present study, a voluntary alcohol intake paradigm was utilized after the individuals had exhibited a stable choice behavior in the rGT, to further examine the relationship between behavior in the rGT and alcohol intake as well as possible alcohol-induced effects on rGT strategies.

Compulsive sexual behavior has high comorbidity with GD and substance use disorders (33–35). Moreover, GD and compulsive sexual behavior are more common in patients with Parkinson's disease treated with dopaminergic therapy than in the general population (36–39). To the best of our knowledge, no study has so far investigated the association between rGT performance and sexual behavior. A study differentiating between motor and choice impulsivity in rats found that impulsivity measures were unrelated to sexual behavior when looking at the number of mounts, intromissions and ejaculations (40). However, sexual behavior consists of both sexual motivation and consummatory components (41, 42) and constitutes a key hedonic behavior. Therefore, the present study investigated how rats with different rGT strategies responded to this natural reward.

There is currently no pharmacological treatment that has a formal indication for GD, but some substances have shown promising results and opioid receptor antagonists, such as naltrexone, are so far the most evaluated and promising (43, 44). Naltrexone is since long registered for the treatment of AUDs (45). Four RCTs have been published on the use of naltrexone in treating GD (46–49), and the results of the two pharmacotherapy-only studies do indicate that naltrexone reduces gambling urges and behaviors (46, 47). To the best of our knowledge, only one previous study has investigated the effects of naltrexone on performance in the rGT and reported improved performance (50). Given the few preclinical studies performed, further investigations are needed.

To further investigate shared and unique features of individual differences in rGT strategies and other reward-related behaviors, the aims of the present experiment were to, in rats with different rGT strategies, explore (I) the stability of rGT strategies over time, (II) motor impulsivity, (III) sexual behavior as a natural reward, and (IV) voluntary alcohol intake and preference. Finally, the response to naltrexone on performance in rats with different rGT strategies was investigated.

## Materials and methods

**Figure 1:**
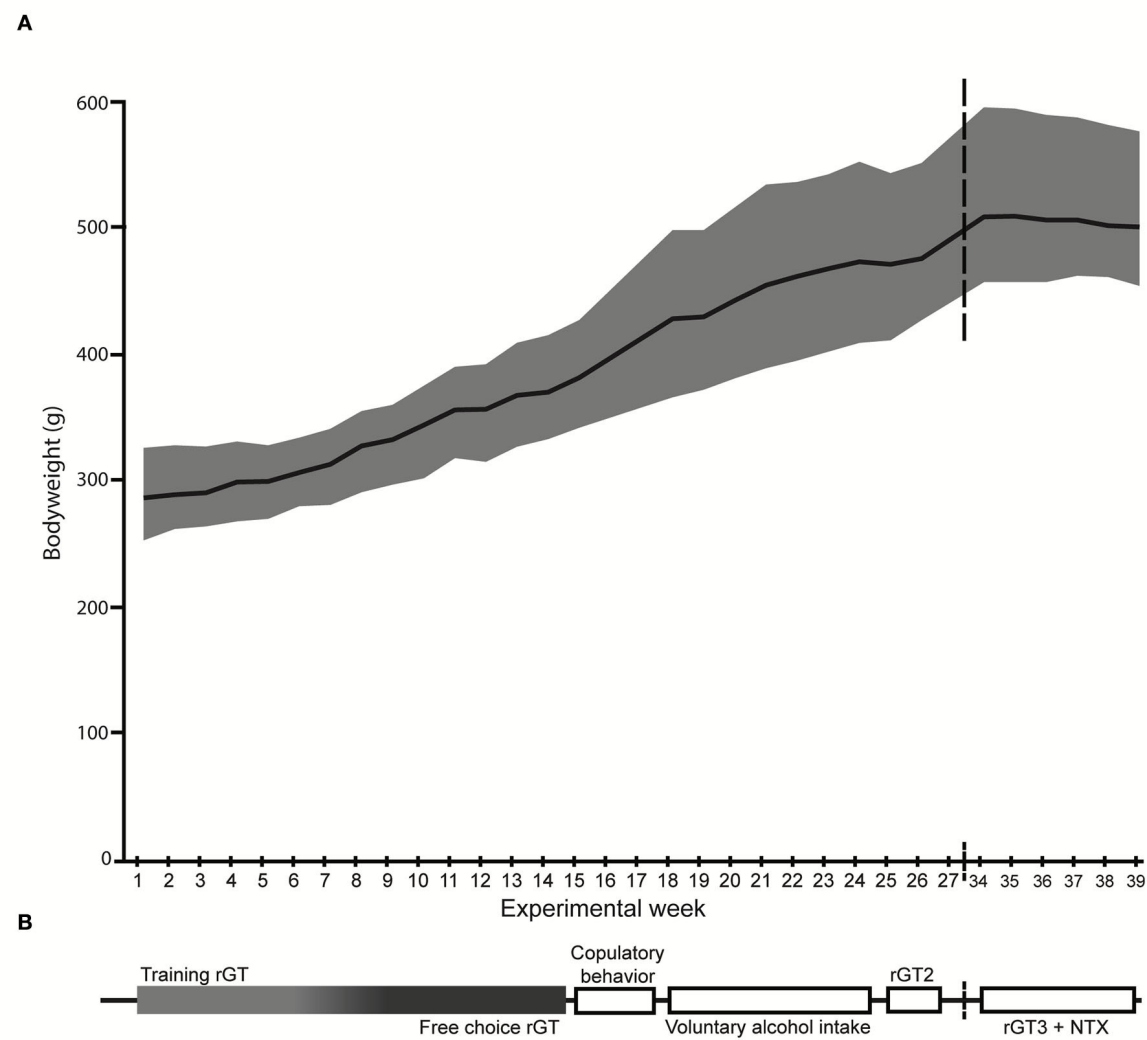
Overview of the experimental procedures. **(A)** The mean body weight (g) is displayed by the black line and the gray shadow shows the body weight range. **(B)** The experimental outline with the order of tests shown, corresponding to the weeks on the *x*-axis in **(A)**. NTX, naltrexone; rGT, rat gambling task.

An overview of the experimental procedures is shown in Figure 1.

### Animals and housing

Male Lister hooded (HsdOla:LH, Envigo, Horst, the Netherlands, *n* = 40) rats were delivered at 6 weeks of age. After arrival, the animals were left undisturbed for 2 weeks in order to acclimatize to the facility and the reversed light/dark cycle (51). Following the acclimatization period, all animals were marked by ear punching. During the week prior to the start of rGT training, all rats were individually handled and weighed in order to habituate to the experimenter. The animals were pair-housed in transparent cages type IV (59 × 38 × 20 cm) with raised lids containing wood chip bedding. For enrichment purposes, each cage had paper sheets (40 × 60 cm, Cellstoff, Papyrus) and a wood tunnel. The cages were kept in an animal room on reversed light/dark cycle (lights off at 6:00 am) with masking background noise. The animal room was kept at a constant temperature (22 ± 1°C) and humidity (50 ± 10%). The animals had access to rat chow (Type R36, Lantmännen, Kimstad, Sweden) *ad libitum* until the start of the rGT. During the rGT, the rats were food restricted to 85% of their free-feeding weight and maintained on 14 g of rat chow given 1 h after their gambling session. During rGT3 the rats had increased in body weight and were given 17.5 g to avoid weight loss. The chow was spread out in the cage in order to secure access for both individuals in a pair. A body weight of the animals was closely monitored (Figure 1A) to ensure that the food restriction was properly carried out. Water was available *ad libitum* during the whole experiment.

Ovariectomized female Long Evans rats (RjOrl:LE, Janvier Labs, Le Genest-st-Isle, France, *n* = 18) were delivered at 11 weeks of age, weighed 189–227 g, and were used as stimuli in the copulatory behavior test. After arrival, the animals were left undisturbed for 2 weeks in order to acclimatize to the facility and the reversed light/dark cycle (51). They were kept 3–4 per cage in the same cage type and environment as the males.

All animal experiments were approved by the Uppsala Animal Ethical Committee (permit number 5.8.18-00833/2017) and followed the guidelines of the Swedish Legislation on Animal Experimentation (Animal Welfare Act SFS 2018:1192) and the European Union Directive on the Protection of Animals Used for Scientific Purposes (Directive 2010/63/EU).

### Rat gambling task (rGT)

The rGT procedure has been described in detail elsewhere (15).

#### Apparatus

The rGT took place in five-hole operant chambers (34 × 33 × 33 cm) placed inside ventilated sound-attenuating cabinets (56 × 56 × 70 cm; Med Associates, Inc., St. Albans, VT, USA). The chambers included response holes, a food tray, and a house light. Both the response holes and the food tray were equipped with stimulus lights and photo beams to record responses. The food tray was connected to a pellet dispenser that delivered 45-mg sucrose pellets (Sandown Scientific, Middlesex, UK). The chambers were controlled by software written in Med PC (Med Associates, Inc.). The chambers were cleaned with 10% ethanol solution and allowed to dry in between subjects.

#### Habituation and training

The rats were habituated to the chambers on two daily 30-min sessions where sucrose pellets were placed in all four nose-poke holes as well as in the food tray. Following this, the rGT training started, and the rats had to progress through six levels of increasing complexity. The training schedule is similar to that for the five-choice serial reaction time task and was based on the schedule published by Zeeb et al. (11) but with some modifications. The last step of the training was a forced-choice rGT that had all the same parameters as the free-choice rGT (described in the following section), with the exception that only one response hole was lit and only a response in that hole gave rise to either a pellet reward or a punishing timeout. This was done for seven sessions to make sure that all the choice alternatives had been explored.

#### rGT

**Figure 2:**
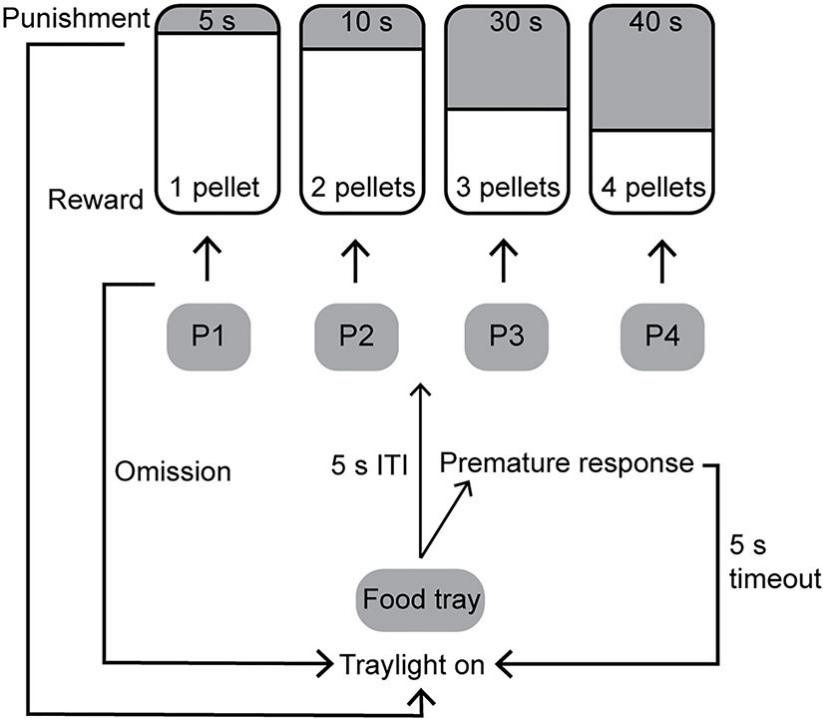
Schematic of the rat gambling task [rGT; (15)]. The contingencies with regard to reward probability, number of pellets, and duration of punishing timeouts for the different options were: P1 *p* = 0.9, 1, and 5 s; P2 *p* = 0.8, 2, and 10 s; P3 *p* = 0.5, 3, and 30 s; P4 *p* = 0.4, 4, and 40 s. ITI, inter-trial interval.

A schematic of the rGT is shown in Figure 2. During the free-choice rGT the rat was able to make a free choice between the four different holes. A trial was initiated by a response in the illuminated food tray. The trial began with a 5-s ITI before the response lights were illuminated and a response could be made. Any response made during the ITI was recorded as a premature response (PR), and the house light was turned on for 5 s before another trial could be started. If no response was made within 10 s after the response holes were activated, the trial was recorded as an omission and the tray light was re-illuminated and a new trial could be initiated. The response holes were associated with a different number of pellets (P), length of punishing timeouts, and probabilities of reward, or punishing timeout (Figure 2). The contingencies with regard to reward probability, number of pellets, and duration of punishing timeouts for the different options were as follows: P1 *p* = 0.9, 1, and 5 s; P2 *p* = 0.8, 2, and 10 s; P3 *p* = 0.5, 3, and 30 s; P4 *p* = 0.4, 4, and 40 s (Figure 2). With these contingencies, the hypothetical number of pellets earned over 30 min was as follows: P1 295, P2 411, P3 135, and P4 99, which makes P2 the most strategic option and P4 the most disadvantageous option.

The task was performed five consecutive days per week, the sessions lasted for 30 min, and the rats could perform as many trials as they wanted during the 30 min. The percentage of each choice was calculated [(#choice of that option/#completed trials) × 100] for P1, P2, P3, and P4. PRs and omissions were recorded as a total number during each session. Additional responses in the response holes after a choice had been made was defined as perseverative responses, divided into perseverative responses during reward and perseverative responses during punishment, and was divided by a number of rewarded or punished trials. Total head entries during punishment, that is, both response holes and the food tray, were also recorded and divided with punished trials.

As shown in Figure 1B, rGT1 was performed immediately following the training period, while rGT2 started at 35 weeks of age after completion of the copulatory behavior tests and the 7 weeks of voluntary alcohol intake, and finally, rGT3 started at 44 weeks of age and continued until the experiment was terminated.

#### Inter-trial interval extension

To add on to our previous findings (15), the present study included an ITI extension in order to assess motor impulsivity. During the first week after training was completed in rGT1, the animals underwent a session with an extended ITI, when the time period that the animals had to wait after starting a trial (Figure 2) was increased to 7 s.

### Naltrexone treatment

During the last 3 weeks of rGT3, naltrexone treatment was given with two doses (0.3 and 3.0 mg/kg) or saline based on previous studies (52–54). Naltrexone hydrochloride (Sigma-Aldrich, Schenndorf, Germany) was dissolved in saline and administered subcutaneously at 1 ml/kg. Naltrexone and saline were administered in a Latin square design (53, 54), so that all animals received both doses as well as saline but not in the same order. Injections were given 30 min prior to the rGT session, followed by four wash-out sessions. Thus, the effects of the treatment on rGT behavior were assessed at 30 min, 24 h, and 48 h following administration.

### Copulatory behavior

The week after rGT1 was completed, the copulatory tests were initiated at 25 weeks of age (Figure 1B). Copulatory behavior was scored in 3 consecutive tests with six test-free days between each session. The females were brought into estrus by hormone treatment consisting of subcutaneous administrations of 25 mg/kg of estradiol benzoate (Sigma-Aldrich, St. Louis, MO, USA) in olive oil 48 h before progesterone and 1 mg/rat of progesterone (Sigma-Aldrich) in olive oil 4–6 h before testing (55). The tests were performed in a wooden cage (60 × 35 × 35 cm) with a transparent front, during the dark phase of the light/dark cycle. The male rat was allowed to habituate to the cage for 5 min before the receptive female was introduced, and thereafter the test lasted for 20 min. Each female was used for two to three males and was alternated during the three copulatory tests. The behavior was live-scored, according to the ethogram in Supplementary Table S1, by an experienced observer who was blind to the performance in the rGT. Additional parameters were calculated based on the scored behaviors.

In the interpretation of male rat sexual behavior, mount and intromission latencies are considered measures of appetitive acts or sexual motivation, while ejaculation represents a consummatory act. Copulatory rate is considered a mixture of sexual motivation and potency, while the interpretation of post-ejaculatory interval is less clear (41, 42).

### Voluntary alcohol intake

At 28 weeks of age, the week after the copulatory tests were completed, the rats received access to alcohol (Figure 1B) through a modified intermittent two-bottle free-choice (20% v/v alcohol solution and water) paradigm with alcohol access for 3 consecutive days per week followed by 4 days of water only (54, 56–58). Dividers were put in before the first 24-h session of the week and taken out after the last session. The dividers are made of transparent plastic with a wire mesh section. The use of dividers provided individual intake measurements during alcohol intake sessions but also allowed the rats some tactile contact (59, 60). Alcohol solution (diluted in tap water from 96% ethanol, Solveco Etanol A 96%; Solveco AB, Rosersberg, Sweden) and tap water was provided in 150-ml bottles with ball valve nipples (Scanbur AB, Sollentuna, Sweden), with minimal spillage. Fresh alcohol solution and water at room temperature were provided for every session, and bottle positions were rotated to avoid any side bias. During the days between the alcohol sessions, the animals were in full social contact and had access to two bottles of tap water. Individual alcohol and water intake were measured every 24 h during the 3 access days for a total of 7 weeks (21 sessions in total). Alcohol intake (g/kg), alcohol preference (% of total fluid intake), water intake (g/kg), and total fluid intake (g/kg) were calculated for each session. To minimize disturbing factors during the intake measures, the cages were changed and animals were weighed on a day with access to water only.

### Statistical analyses

Statistical analyses were carried out in Statistica 13 (TIBCO Software, Inc., Tulsa, OK, United States) unless otherwise specified. Data were considered statistically significant at *p* < 0.05. Parameters were examined for normality using the Shapiro–Wilk's *W*-test. The majority of all parameters in the rGT, including the effects of naltrexone treatment, as well as copulatory behavior and voluntary alcohol intake, were not normally distributed; hence, non-parametric statistics were used. Analyses of main effects and interactions in non-parametrical, longitudinal data sets from the copulatory behavior tests, voluntary alcohol intake, and naltrexone treatment were carried out in R 4.0.2 (61) using the nparLD package (62). For the copulatory behavior tests, the rGT strategy group was used as a between-subject factor, and testing day as a within-subject factor. The npardLD package requires complete data sets without missing values, and therefore, some parameters from the copulatory behavior tests had to be excluded from the analysis (as described in Supplementary Table S5). For the voluntary alcohol intake, rGT strategy group was used as a between-subject factor, and drinking week as a within-subject factor. For the naltrexone treatment, rGT strategy group was used as between-subject factor, and dose and time after treatment as within-subject factors. Between-subject *post hoc* tests were performed with the Mann–Whitney *U*-test with continuity correction (voluntary alcohol intake), and within-subject *post hoc* tests were performed with the Wilcoxon's matched pairs test (copulatory behavior tests, naltrexone treatment). For the remaining parameters from the copulatory tests as well as for ITI extension differences over time were analyzed with Friedman ANOVA followed by Wilcoxon matched-pairs tests where appropriate. Individual stability over rGT1–3 was investigated using the Spearman rank-order correlations between sequential occasions (rGT 2 vs. 1, rGT 3 vs. 2, and rGT3 vs. 1) (63).

## Results

### rGT1

**Figure 3:**
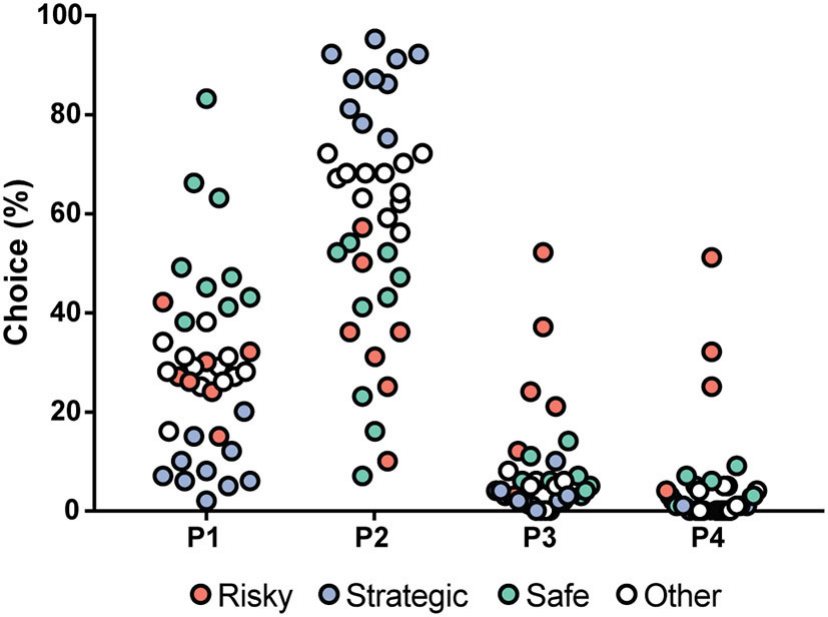
Distribution of the choices (%) in all rats (*n* = 40) tested during rGT1, with choices in percent on the *y*-axis and the four available choices on the x-axis. The individuals are colored by rGT strategy group into safe (green, *n* = 9), strategic (blue, *n* = 10), and risky (red, *n* = 7). The safe individuals had a high percentage of choices of P1, the strategic individuals had a high percentage of choices of P2, and the risky individuals had a high percentage of choices of P3 and P4. The un-colored dots (other, *n* = 12) represent all individuals who were not a part of the group being displayed in any choice category.

The choices in the rGT1 for all rats during each week are shown in Supplementary Table S2. During the first week, P1 was the most frequently chosen option (57%), but during Week 2, P2 became the most chosen option and remained the most chosen option for the group of rats as a whole during the rest of the rGT1. The progression of choices is shown in Supplementary Figure S1. The rGT strategies started to become visible during Week 2 and remained stable for the rest of rGT1. Based on the patterns and according to a previous study (15), rGT strategy groups were formed based on the distribution of choices during Week 5 (Figure 3). The top quartile in P1 formed the safe group, and the top quartile in P2 formed the strategic group. The risky group included the individuals with P3% > Q3 + 1.5 ^*^ IQR (interquartile range) and P4% > Q3 + 1.5 ^*^ IQR. The remaining individuals constituted the other group (Figure 3, Supplementary Figure S1).

### ITI extension

**Figure 4:**
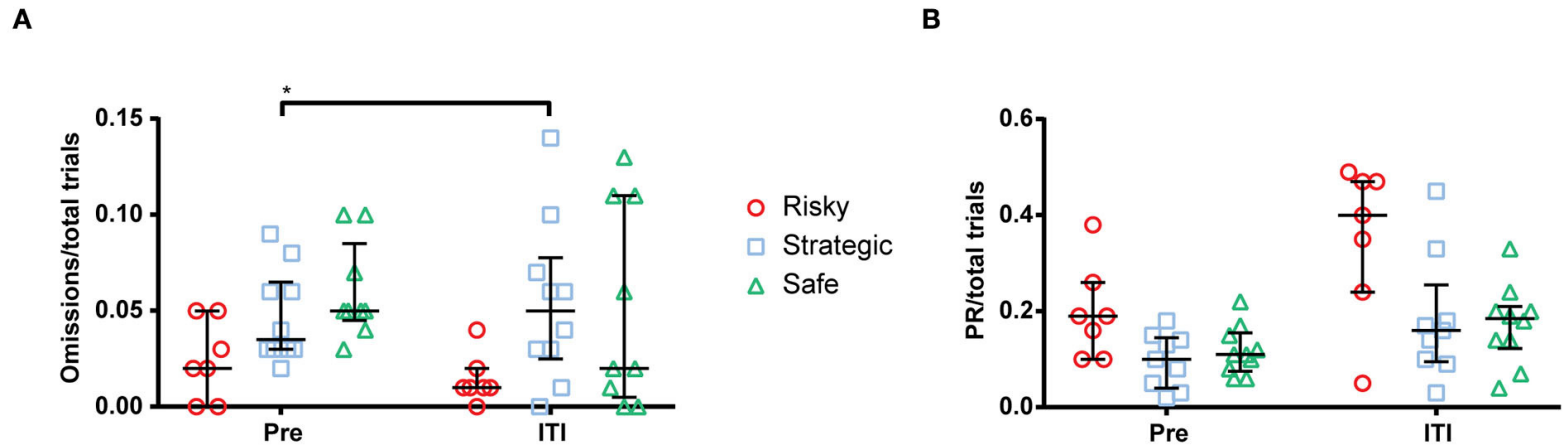
Number of **(A)** omissions per total trial and **(B)** premature responses (PR) per total trial during a mean of 3 days prior to (pre) and during the extended inter-trial interval (ITI) of 7 s. *< 0.05 (*post hoc* Wilcoxon matched-pairs test).

The ITI extension was performed during one session in the first week of rGT1 and compared to the mean performance during 3 days prior to the extended ITI (pre). The results from the ITI extension on rGT parameters are shown in Supplementary Table S3. All groups made fewer total trials and fewer completed trials during the extended ITI (Supplementary Table S3). Moreover, the strategic rats increased their omissions/total trials (Figure 4A), and the choice of P2 increased, and the choice of P3 decreased during the extended ITI (Supplementary Table S3), while PRs/total trial was statistically unaffected but numerically highest in the risky rats (Figure 4B). The extended ITI had no effect on choices made by risky or safe rats (Supplementary Table S3).

### rGT2–3

**Figure 5:**
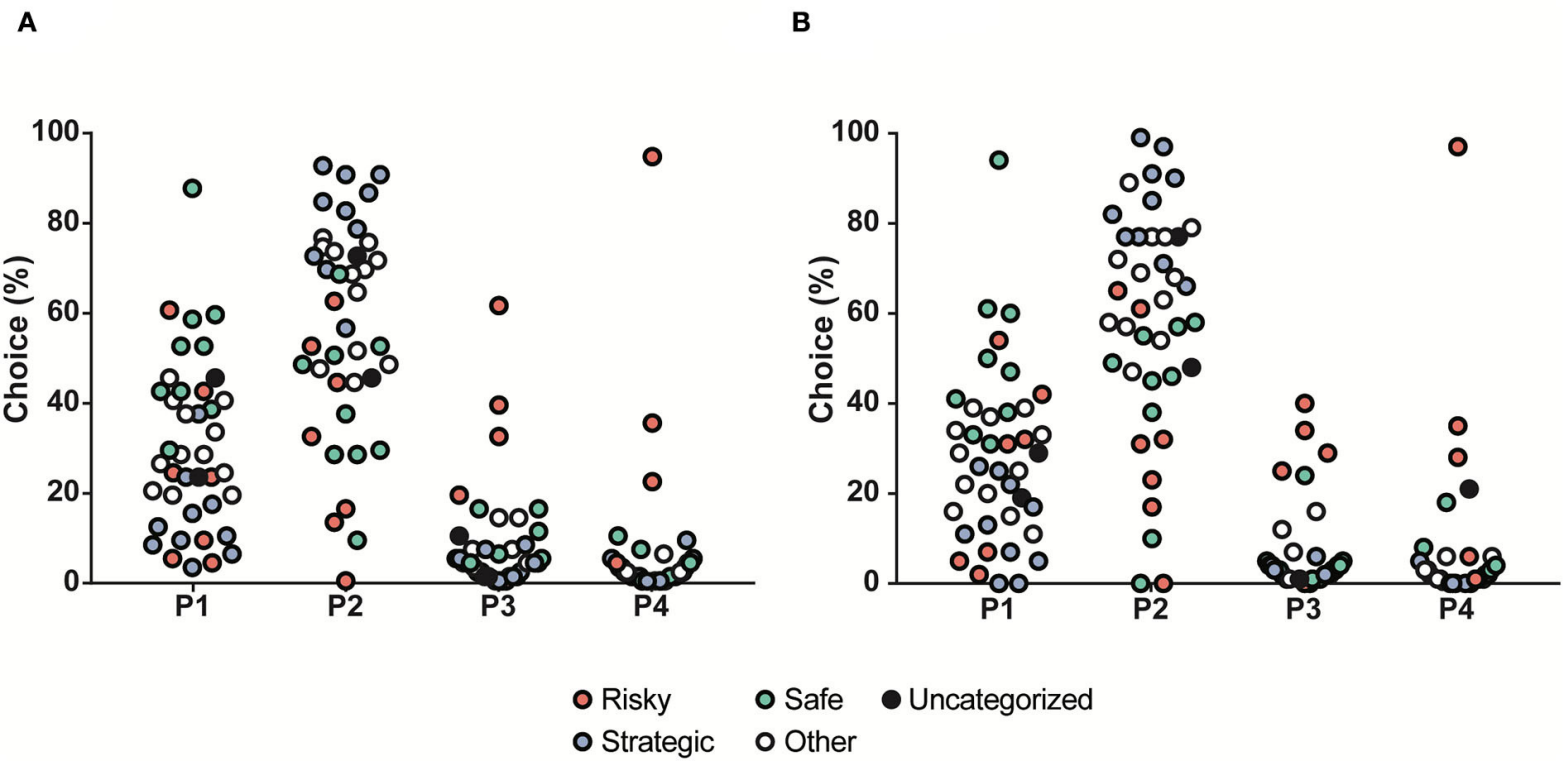
Distribution of the choices (%) in all rats (*n* = 40) tested during **(A)** rGT2 and **(B)** rGT3, with choices in percent on the *y*-axis and the four available choices on the *x*-axis. The individuals are colored by the rGT group in rGT1 into safe (green, *n* = 9), strategic (blue, *n* = 10), risky (red, *n* = 7), other (white, *n* = 12), and individuals that did not advance past training in rGT1 (black, *n* = 2).

The second round of rGT lasted for 2 weeks. Even though this rGT period was short and the animals had undergone a 7-week interruption with testing in other tests, while under *ad libitum* feeding, the choice patterns were similar to during rGT1 (Figure 5A). Following another interruption of 7 weeks, the rats underwent rGT3 for 3 weeks prior to naltrexone treatment. The data presented for rGT3 (Figure 5B) comprise the last 3 days of the third week and reveal that the same stable rGT pattern remained. The stability is demonstrated by correlations between choices in rGT1, rGT2, and rGT3 (Supplementary Table S4). Some individuals did not reach past the training part during rGT1; they were still included in rGT2 and rGT3 but were not included in any rGT strategy group, illustrated in the figures with black symbols (Figure 5).

The choice groups were re-calculated based on the results from rGT3 for analysis of the effects of naltrexone treatment on rGT parameters. Several of the rats changed strategy group compared to rGT1, 14% in the risky group, 30% in the strategic group, 44% in the safe group, and 50% in the other group.

### Sexual behavior

Results from the copulatory behavior tests for all rats irrespectively of rGT strategy are shown in Supplementary Table S5. Analyses of main effects and interactions revealed that there was only an effect of test in performance [latency mount (ATS = 13.5, df = 1.7, *p* < 0.001), latency intromission (ATS = 21.5, df = 1.6, *p* < 0.001), latency ejaculation (ATS = 21.1, df = 1.9, *p* < 0.001), frequency mount (ATS = 6.1, df = 2.0, *p* < 0.01), frequency intromission (ATS = 3.9, df = 1.9, *p* < 0.05), frequency ejaculation (ATS = 12.1, df = 1.9, *p* < 0.001), and mounts + intromissions (ATS = 8.0, df = 1.9, *p* < 0.001)], and no effect of rGT strategy group or interaction between test and group was found. Performance among all rats increased over time, as indicated by shorter latencies and higher frequencies of mounts, intromissions, and ejaculations over tests. Moreover, time from the first intromission to ejaculation and inter-intromission rate decreased over time, and finally, copulatory rate increased over time (Supplementary Table S5). Post-ejaculatory interval, time from first intromission to ejaculation and copulatory rate in the different rGT strategy groups are shown in Supplementary Figure S2.

### Alcohol intake

**Figure 6:**
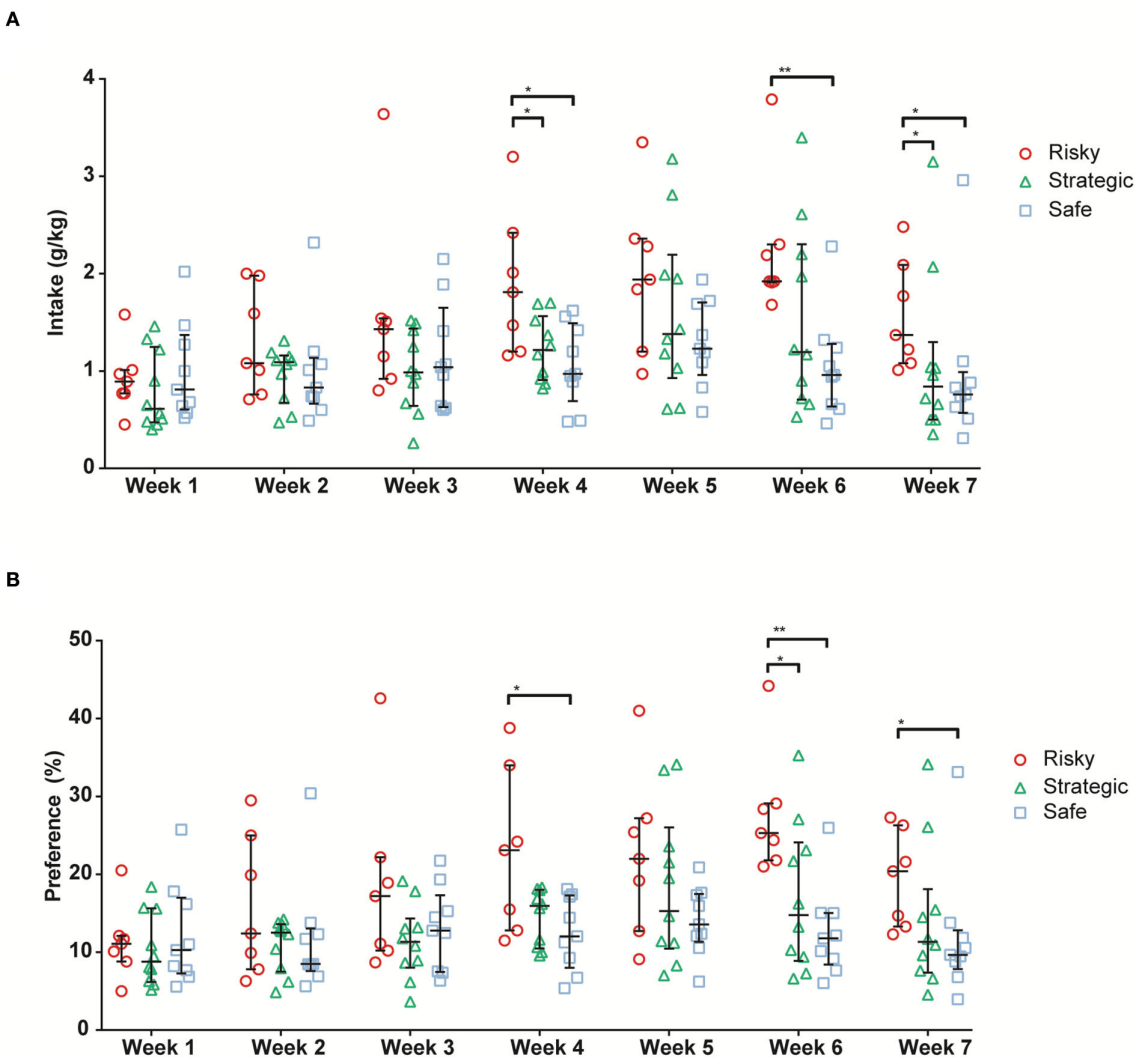
Average alcohol intake [g/kg; **(A)**] and alcohol preference [%; **(B)**] during the 7 weeks of alcohol access in rats with risky (*n* = 7), strategic (*n* = 10), and safe (*n* = 9) rGT strategies. Data are presented as individual rats with group median and quartile range marked. * <0.05, ** <0.01 (*post hoc* Mann–Whitney *U*-test).

Individual alcohol and water intake were measured every 24 h during the 3 consecutive access days for a total of 7 weeks (21 sessions in total). The main effect of time was seen for alcohol intake (ATS = 12.8, df = 4.6, *p* < 0.001), alcohol preference (ATS = 10.0, df = 4.5, *p* < 0.001), water intake (ATS = 6.0, df = 4.7, *p* < 0.001), and total fluid intake (ATS = 7.4, df = 4.5, *p* < 0.001). A main effect of rGT strategy group was seen for alcohol intake (ATS = 3.3, df = 2.0, *p* < 0.05). Moreover, an interaction between time and the rGT strategy group was seen for alcohol intake (ATS = 2.3, df = 7.3, *p* < 0.05) and alcohol preference (ATS = 2.2, df = 7.1, *p* < 0.05). The alcohol intake and preference are shown in Figure 6. The risky rats had a higher voluntary alcohol intake than the strategic and safe rats during Weeks 4 and 7 and higher intake than the safe rats during Week 6 (Figure 6A). The differences in preference were similar; the risky rats had higher alcohol preference than the safe rats during Weeks 4 and 7 and higher preference than the strategic and safe rats during Week 6 (Figure 6B). No differences between the groups in water intake (Supplementary Table S6A) or total fluid intake (Supplementary Table S6B) were found. Finally, the 7 weeks of voluntary alcohol intake had no effect on the preceding rGT behavior in any of the groups during rGT2.

### Naltrexone treatment

**Figure 7:**
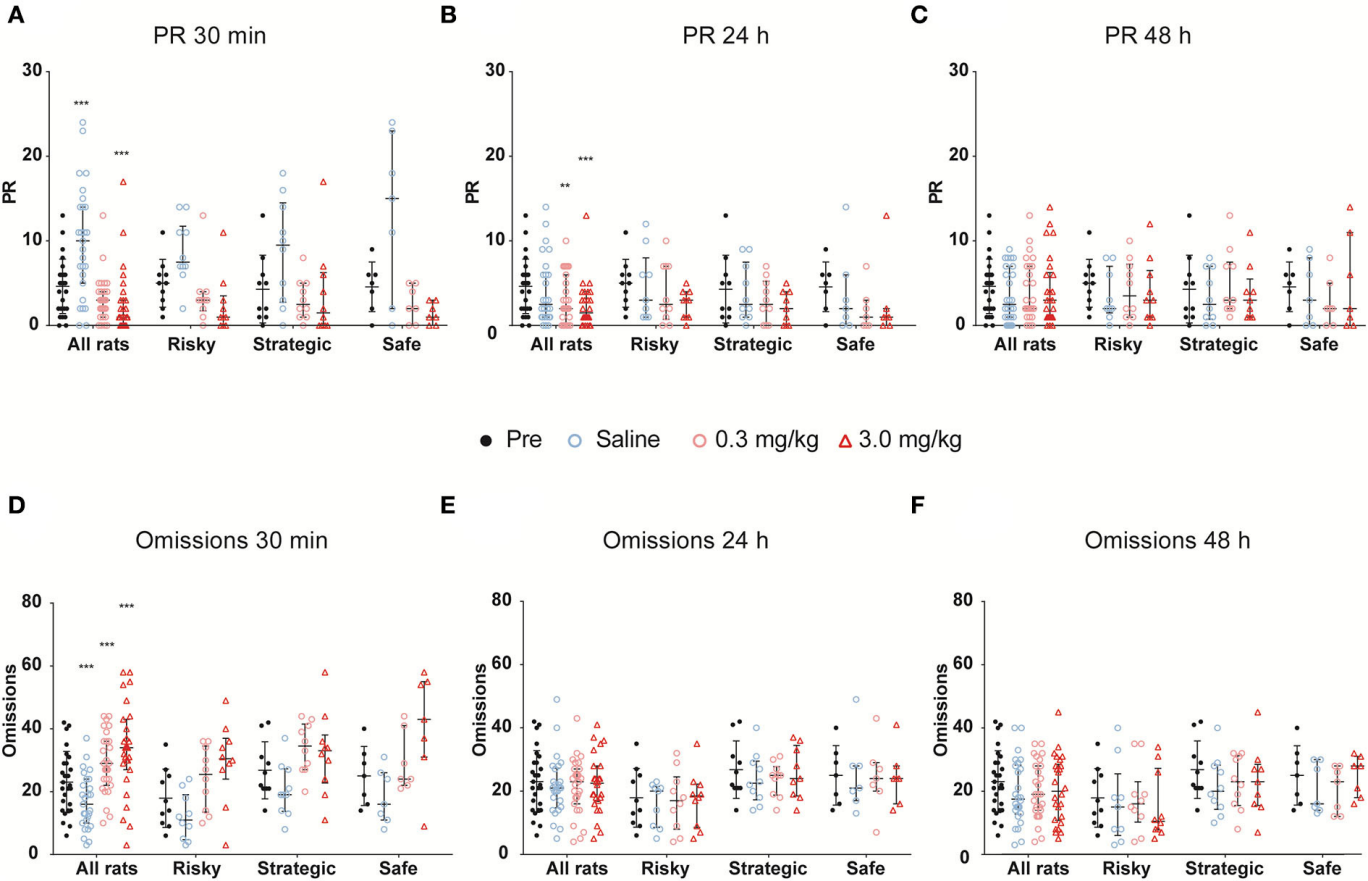
The effects of naltrexone on **(A–C)** premature responses (PRs) and **(D–F)** omissions when assessed **(A,D)** 30 min, **(B,E)** 24 h, and **(C,F)** 48 h after treatment with naltrexone (0.0, 0.3, and 3.0 mg/kg) in all rats irrespectively of rGT strategy as well as in rats with risky (*n* = 7), strategic (*n* = 10), and safe (*n* = 10) rGT strategies. Each figure contains the data prior to treatment (pre, mean of 3 days prior) as a baseline comparison. Data are presented as individual rats with group median and quartile range marked. ** < 0.01, *** < 0.001 compared to pre-treatment in all rats (*post hoc* Wilcoxon matched-pairs test).

After 3 weeks of testing in rGT3, naltrexone (0.0, 0.3, and 3.0 mg/kg, s.c.) was administered in a Latin square design 30 min prior to rGT. The statistical results from the omnibus tests are shown in Table 1. Omissions, PR, total trials, and number of completed trials were among the parameters with a significant main effect of dose, as well as an interaction between dose and time (Table 1). The results for all rats irrespectively of rGT strategy are shown in Figure 7, Supplementary Table S7. In all rats irrespectively of rGT strategy, saline increased, and naltrexone at both doses decreased the number of completed trials and total trials, respectively, at 30 min, with the higher dose of naltrexone also decreasing the number of completed trials and total trials at 24 h (Supplementary Table S7). Moreover, saline increased and naltrexone at the higher dose decreased PR at 30 min (Figure 7A, Supplementary Table S7), while both doses of naltrexone decreased PR at 24 h (Figure 7B, Supplementary Table S7). Finally, saline decreased, and naltrexone at both doses increased the number of omissions at 30 min (Figure 7D, Supplementary Table S7). No effect of naltrexone was evident 48 h after administration for omissions, PR, total trials, and number of completed trials (Figure 7, Supplementary Table S7).

**Table 1:** Statistical main effects and interactions on rGT parameters after naltrexone treatment.

**Parameter**	**Main effects**	**Interactions**
Omissions	Dose (ATS = 14.5, df = 1.9, *p < * 0.001) Time (ATS = 5.2, df = 2.4, *p < * 0.01)	Dose x Time (ATS = 13.2, df = 4.0, *p < * 0.0001)
Premature responses	Dose (ATS = 9.2, df = 2.0, *p < * 0.001) Time (ATS = 4.3, df = 2.9, *p < * 0.01)	Dose x Time (ATS = 6.3, df = 4.0, *p < * 0.001)
Perseverative responses during punishment	Dose (ATS = 5.7, df = 1.7, *p < * 0.01) Group (ATS = 5.0, df = 1.9, *p < * 0.01)	Dose x Time (ATS = 6.3, df = 4.4, *p < * 0.001)
Perseverative responses during reward	Time (ATS = 5.3, df = 2.8, *p < * 0.01)	Group x Time (ATS = 2.5, df = 4.9, *p < * 0.05)
Head entries	Dose (ATS = 11.5, df = 1.6, *p < * 0.001) Group (ATS = 8.8, df = 1.7, *p < * 0.001)	Dose x Time (ATS = 5.8, df = 3.6, *p < * 0.001)
Total trials	Dose (ATS = 20.0, df = 1.8, *p < * 0.001) Group (ATS = 3.2, df = 1.8, *p < * 0.05)	Dose x Time (ATS = 24.5, df = 3.6, *p < * 0.001)
Completed trials	Dose (ATS = 41.7, df = 1.7, *p < * 0.001) Time (ATS = 6.5, df = 2.3, *p < * 0.001)	Dose x Time (ATS = 50.0, df = 3.7, *p < * 0.001)
P1%	Group (ATS = 26.0, df = 1.8, *p < * 0.001) Time (ATS = 11.7, df = 2.4, *p < * 0.001)	Dose x Time (ATS = 3.4, df = 4.2, *p < * 0.01)
P2%	Group (ATS = 23.5, df = 1.9, *p < * 0.001) Time (ATS = 12.6, df = 2.3, *p < * 0.001)	Dose x Time (ATS = 3.1, df = 4.1, *p < * 0.05)
P3%		
P4%	Group (ATS = 5.2, df = 1.7, *p < * 0.01) Time (ATS = 3.2, df = 2.6, *p < * 0.05)	

Main effects and interactions of time (pre, 30 min, 24 h, and 48 h after administration of naltrexone), dose of naltrexone (0.0, 0.3, and 3.0 mg/kg), and rGT group [risky (n = 7), strategic (n = 10), and safe (n = 10)] on rGT parameters. ANOVA-type statistics (ATS) according to the R package nparLD (62).

Parameters that had the main effect of rGT strategy group were perseverative responses during punishment, head entries, total trials, as well as P1, P2, and P4% (Table 1). In the risky group, saline increased perseverative responses during punishment at 30 min, and the high dose of naltrexone decreased perseverative responses during punishment at 30 min and 24 h after administration (Supplementary Table S7). Moreover, the number of head entries was decreased at the high dose at both 30 min and 24 h, as well as at 30 min after the lower dose of naltrexone (Supplementary Table S7). Finally, saline increased, and naltrexone at both doses decreased total trials at 30 min, with the higher dose of naltrexone also decreasing total trials at 24 h (Supplementary Table S7). In the strategic group, saline increased, and naltrexone at the higher dose decreased total trials at 30 min and 24 h (Supplementary Table S7). Moreover, P1% was decreased at 24 h following the high dose of naltrexone (Supplementary Table S7). Finally, saline decreased P2% at 30 min and 24 h, with a decrease at 30 min also following the low dose of naltrexone. In the safe group, P2% decreased 30 min after saline as well as both the low and high doses of naltrexone (Supplementary Table S7).

## Discussion

The novel results of this study demonstrate that individual differences in rGT strategies were stable over time. Moreover, rats with risky rGT strategies had higher voluntary alcohol intake and preference and higher motor impulsivity but not altered sexual behavior; the latter used to investigate the response to a natural reward. Finally, in all rats irrespectively of rGT strategy, naltrexone decreased the number of completed trials and PR as well as increased the number of omissions, indicative of reduced motivation.

In rGT1, individual strategies started to become visible during the third week and remained stable throughout the experiment. Based on the distribution of choices during Week 5, three strategy groups could be formed, that is, safe strategic, and risky, which replicates the findings from our previous study (15). When looking at all rats irrespectively of rGT strategy, the preferred choice was P2, that is, the most strategic choice. This is in line with previous publications using the same rGT version (11, 13–16). When other versions of the rGT were used, the strategic choice was usually the most preferred, but comparisons are more difficult to make since the outline of the test differs in various ways [e.g., (31, 64–67)]. However, in support of the robustness of the test, stable choice preferences appear independent of the version of rGT used.

To the best of our knowledge, this is the first time that repeated interruptions have been used in order to assess stability in gambling strategies over time, that is, the rGT was performed twice more in the present study. In rGT2 and rGT3, similar choice patterns as in rGT1 were found, even though the rGT2 period was short and the animals had undergone testing in other tests since rGT1. When the choice groups were re-calculated in rGT3, several of the rats changed strategy group; 14% in the risky group, 30% in the strategic group, 44% in the safe group, and 50% in the other group. However, the individuals that switched groups were the ones that were close to the “break point” between strategy groups, and most changes occurred to and from the other group. Even though some fluctuation in the exact percentage of the different choices occurred between the three rounds of rGT, the individual choice patterns stayed consistent, and choices of P1, P2, P3, and P4 in the three rounds of rGT were correlated. Notably, the most stable pattern was that of the risky rats. This means that individual choices in the rGT remain stable over time also with rGT interruptions and the experience of other stimuli, herein sexual activity and alcohol intake.

In the rGT, the ITI extension enables investigation of the relationship between choice and motor impulsivity. Here, it was revealed that the risky group, with elevated choice impulsivity, made numerically, but not significantly, more PRs than the strategic and safe rats, indicating higher motor impulsivity. Several contradicting studies have been published, with some that have failed to find a correlation between motor and choice impulsivity in rats (68, 69) and some that have found a correlation (13, 70). The results from the present study thus agree with the latter category, as individuals that made risky choices made more PRs. In previous studies using the rGT, no associations between motor impulsivity and decision-making were revealed (11, 23, 24). However, a recent meta-analysis of 13 experimental cohorts demonstrated a negative correlation between advantageous choices and PRs (13), which agrees with the results in the present study, where the risky rats increased numerically in PRs when challenged with the ITI extension. The number of omissions were also affected by the ITI extension. Omissions in the risky group decreased while the strategic rats increased their omissions, that is, they declined to make a choice when experiencing a change in “rules” of the test. Omissions are usually interpreted as a lack of motivation (71–73), and a relationship between task difficulty and omissions has been found and interpreted as attentional lapses (74). The fact that the risky rats increased the number of PRs while strategic rats increased the number of omissions indicates that the strategic and risky individuals had opposite responses to this challenge.

The rats with risky rGT strategies had a higher voluntary alcohol intake and preference relative to the safe and strategic rats, but no alcohol-induced effects on subsequent gambling in rGT2 were revealed. These findings contrast a previous study using a similar version of the rGT, where it was demonstrated that high alcohol-drinking rats showed more optimal decision-making in the rGT and reduced choice impulsivity in the delayed reward task and that alcohol administration increased optimal decision-making in the rGT in both low and high-drinking rats (32). However, the fact that the rats with a risky rGT strategy also consumed more alcohol is novel and notable from the perspective that GD and AUDs share many features (2, 3) and are highly comorbid (25, 26). A recent meta-analysis revealed that decision-making deficits in the IGT were associated with both AUDs and GD and were more pronounced in GD than in AUDs (27). Moreover, the finding of both high choice and motor impulsivity, as well as a high-alcohol intake in the risky rats agrees with studies showing that animals that have been classified as highly motor impulsive showed an escalation of cocaine intake (75), and showed higher breakpoints under progressive ratio schedules for cocaine (76). However, our findings contradict previous findings in rats where no correlation between motor or choice impulsivity and alcohol intake or motivation for alcohol self-administration was found (77). Studies on the relationship between rGT strategies and alcohol intake in rats are not abundant, and this discrepancy in results indicates that more research is needed.

No differences in sexual behavior, either in appetitive or consummatory components, were revealed in rats with different rGT strategies. A notable observation was that the rats in the present study performed remarkably better on all measures in the copulatory tests than alcohol-preferring sP and outbred Wistar rats in a previous study (55). The copulatory tests were included in the present study to investigate the response to a natural reward due to the shared features between GD and compulsive sexual behavior in humans (78–80). Moreover, some studies have reported higher levels of impulsivity in patients with compulsive sexual behavior (81–84). The present results do not support an association between risky rGT strategies and increased sexual behavior. In support of that finding is a study differentiating between motor and choice impulsivity in rats that found that impulsivity measures were unrelated to sexual behavior (40).

In all rats irrespectively of rGT strategy, naltrexone decreased the number of completed trials and PR as well as increased the number of omissions in the rGT, which indicate a lowered overall motivation. In agreement, decreased number of completed trials was found following naltrexone administration (0.3 mg/kg) in rats performing the delay discounting task (85). These results contrast findings in healthy volunteers reporting recreational gambling, where naltrexone administration did not result in attenuated responses to winning outcomes in a slot machine and Roulette task, respectively (86). To our knowledge, no studies have investigated the effects of naltrexone on outcomes in the IGT. However, the use of naltrexone deserves further investigation given that, in GD, the treatment is promising and able to reduce gambling symptoms when several different scales were used to measure gambling symptoms (44).

In the respective rGT groups, naltrexone administration did not affect the choices in any clear and uniform way. A study investigating the effects of naltrexone treatment in rats using the same rGT as the present but categorizing the animals into advantageous choices (high percentage of P1 and P2) and non-advantageous choices (high percentage of P3 and P4) showed that rats that made fewer advantageous choices at baseline increased their advantageous choices when treated with naltrexone. When looking at the specific choices, the rats increased the choice of P1, but no effect was seen on any other choice (50). In the present study, the safe group decreased in P2 in favor of an indication toward an increase in P1, both considered advantageous, while no attenuation of risky options was observed. The limited number of rats in each group may have affected the power of detecting significant effects of naltrexone within the respective rGT groups.

In conclusion, this study found stable individual choice strategies in the rGT, which replicates our previous findings (15). These choice strategies were stable over time, even after multiple interruptions and behavioral testing in between. The rats with a risky rGT strategy displayed higher motor impulsivity and higher voluntary alcohol intake than the other groups. No difference in sexual behavior was found in the different rGT groups. Finally, treatment with naltrexone showed promising results since the number of completed trials and PR decreased while omissions increased, which indicates a lowered motivation. Given that rats with risky rGT strategies, characterized by altered connectivity in brain reward-related networks (15), had higher voluntary alcohol intake but not elevated sexual behavior, underlying mechanisms between rGT strategies and alcohol intake may be shared, while this is not the case for risky rGT strategies and natural rewards in terms of sexual behavior.

## Data availability statement

The raw data supporting the conclusions of this article will be made available by the authors, without undue reservation.

## Ethics statement

The animal study was reviewed and approved by Uppsala Animal Ethical Committee (permit number 5.8.18-00833/2017) and followed the guidelines of the Swedish Legislation on Animal Experimentation (Animal Welfare Act SFS 2018:1192) and the European Union Directive on the Protection of Animals Used for Scientific Purposes (Directive 2010/63/EU).

## Author contributions

NT collected most of the data, analyzed the data, and wrote the first draft of the manuscript. ER designed the experiment, supervised the experimental execution, data analysis, manuscript outline, and provided the funding. Both authors contributed to manuscript editing and approved the final version of this manuscript.
