# Supplementary material for: Individual strategies in the rat gambling task are related to voluntary alcohol intake, but not sexual behavior, and can be modulated by naltrexone
Source: Front Psychiatry. 2022 Dec 8;13:931241. doi: 10.3389/fpsyt.2022.931241 (PMC9772284; doi:10.3389/fpsyt.2022.931241)
Supplement: Supplementary file 1 [file Data_Sheet_1.docx]

Supplementary Material

# Supplementary Figures

**
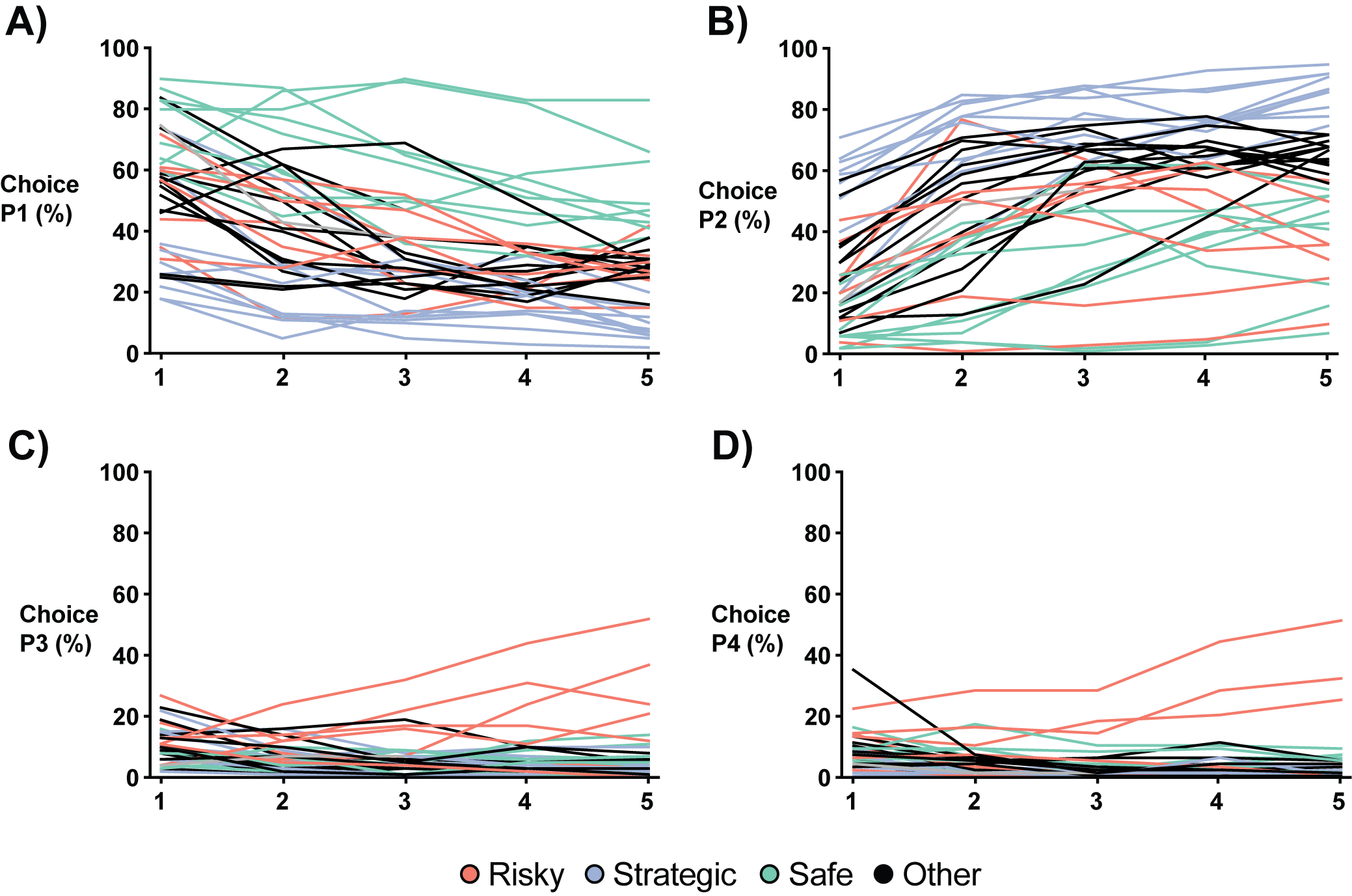
**

**Figure S1.** Individual progression of choices (%) of (A) P1, (B) P2, (C) P3 and (D) P4 during the five weeks of rGT1, with percent of choice on the y-axis and week on the x-axis. The color of the lines indicates rGT group according to the rGT performance during week 5 (Figure 3).


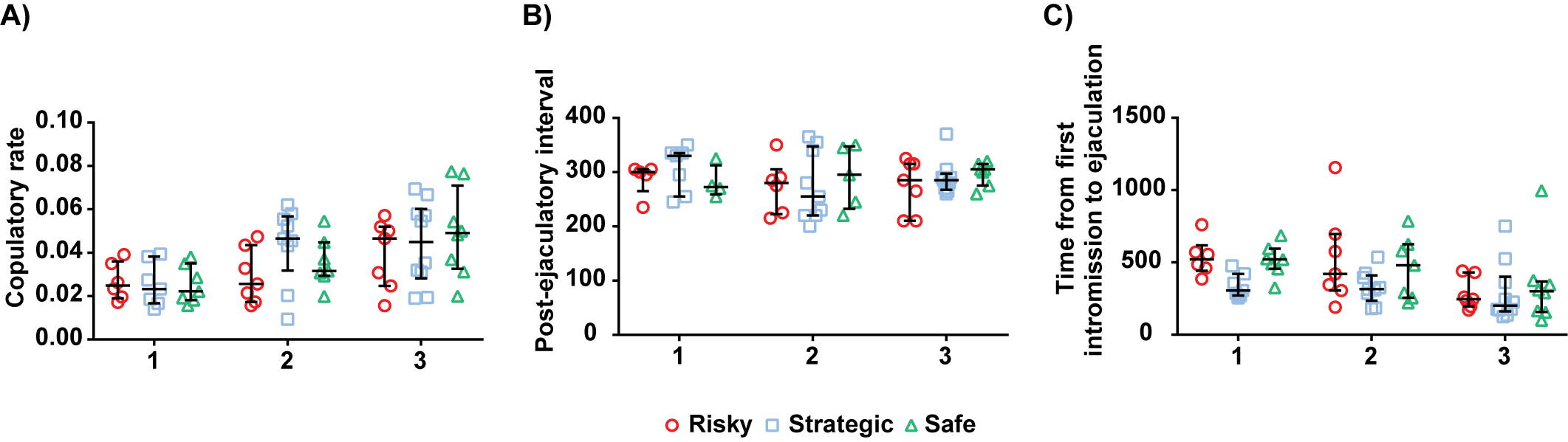


**Figure S2.** Results from the three copulatory behavior tests for (A) copulatory rate, (B) post-ejaculatory interval (s), and (C) time from first intromission to ejaculation (s) in rats with risky (n = 7), strategic (n = 10) and safe (n = 9) rGT strategies. The behaviors are explained in Table S1. Data are presented as individual rats with group median and quartile range marked. No significant differences between the rGT groups were found.

# Supplementary Tables

**Table S1.** Ethogram of the behaviors scored during the copulatory behavior tests.

| Parameter | Definition |
| --- | --- |
| Latency mount | The time (s) from the introduction of the receptive female until first mount |
| Latency intromission | The time (s) from the introduction of the receptive female until first intromission |
| Latency ejaculation | The time (s) from the introduction of the receptive female until first ejaculation |
| Frequency mount | The total number of observed mounts |
| Frequency intromission | The total number of observed intromissions |
| Frequency ejaculation | The total number of observed ejaculations |
| Post-ejaculatory interval | The time (s) from ejaculation until the next observed intromission |
| Mounts + intromissions | The sum of observed mounts and intromissions |
| Intromission ratio | The number of observed intromissions calculated in relation to the total number of observed mounts and intromissions |
| Time from first intromission to ejaculation | The elapsed time (s) from the first observed intromission until an observed ejaculation |
| Mounts + intromissions/ejaculation | The sum of mounts and intromissions required to reach ejaculation |
| Inter-intromission rate | The latency (s) from the introduction of the receptive female until ejaculation divided by the total number of intromissions |
| Copulatory rate | The sum of mounts and intromissions divided by the elapsed time (s) from the first mount until an observed ejaculation |

**Table S2.** Choices in the rGT1 for all rats (n=40) during all weeks.

|  | **n** | **P1%** | **P2%** | **P3%** | **P4%** |
| --- | --- | --- | --- | --- | --- |
| Week 1 | 40 | 57.0 (34.6–70.9) | 24.0 (12.1–42.4) | 9.4 (6.1–12.8) | 6.8 (3.7–9.4) |
| Week 2 | 39 | 43.4 (26.8–58.8) | 48.8 (28.0–66.7) | 6.0 (3.3–9.2) | 2.3 (0.9–5.7) |
| Week 3 | 39 | 30.8 (22.5–47.2) | 61.1 (43.8–69.2) | 4.8 (3.1–7.2) | 1.4 (0.7–3.5) |
| Week 4 | 38 | 26.5 (19.1–35.9) | 62.0 (45.4–74.6) | 4.8 (3.0–9.4) | 1.6 (0.4–4.4) |
| Week 5 | 38 | 27.3 (13.4–39.1) | 62.2 (40.9–85.1) | 3.5 (1.0–7.1) | 1.4 (0.0–5.7) |

Percentage of choice of P1, P2 P3 and P4 in the population of rats during the 5 weeks of rGT1. Data are presented as median and quartile range.

**Table S3.** rGT parameters prior to (pre; mean of three preceding days) and during the extended inter-trial interval (ITI) in rats with risky, strategic and safe rGT strategies.

| **Parameter** |  | **Risky (n = 7)** | **Strategic (n = 10)** | **Safe (n = 9)** |
| --- | --- | --- | --- | --- |
| Omissions | Pre ITI | 2.7 (0.3–4.7) | 4.8 (3.7–6.7) | 5.7 (5.3–8.3) |
|  | Extended ITI | 1.0 (1.0–2.0) | 5.0 (3.0–7.0) | 3.0 (1.0–9.0) |
| Omissions/total trials | Pre ITI | 0.0 (0.0–0.1) | 0.0 (0.0–0.1) | 0.1 (0.1–0.1) |
|  | Extended ITI | 0.0 (0.0–0.0) | 0.2* (0.1–0.4) | 0.0 (0.0–0.1) |
| Premature responses | Pre ITI | 17.0 (15.7–36.7) | 12.2 (8.7–14.3) | 12.7 (5.3–16.7) |
|  | Extended ITI | 38.0 (29.0–55.0) | 20.5 (13.0–23.0) | 17.0 (11.0–21.0) |
| PR/total trials | Pre ITI | 0.2 (0.1–0.3) | 0.1 (0.1–0.2) | 0.1 (0.1–0.2) |
|  | Extended ITI | 0.7 (0.3–0.9) | 0.2 (0.2–0.3) | 0.2 (0.1–0.2) |
| PRdP | Pre ITI | 83.3 (64.0–116.0) | 64.8 (51.7–74.0) | 56.0 (47.7–65.0) |
|  | Extended ITI | 81.0 (68.0–84.0) | 33.5** (26.0–53.0) | 41.0 (37.0–52.0) |
| PRdR | Pre ITI | 6.0 (4.0–17.0) | 1.7 (1.3–4.7) | 3.0 (2.0–4.0) |
|  | Extended ITI | 3.0 (2.0–7.0) | 0.5 (0.0–4.0) | 1.0 (1.0–2.0) |
| Head entries | Pre ITI | 23.7 (23.7–79.0) | 38.8 (27.3–48.0) | 20.3 (15.7–24.7) |
|  | Extended ITI | 26.0 (19.0–47.0) | 29.0* (6.0–34.0) | 10.0 (7.0–24.0) |
| Total trials | Pre ITI | 88.3 (79.7–105.3) | 94.5 (89.7–123.0) | 105.7 (99.7–108.0) |
|  | Extended ITI | 63.0 (54.0–83.0) | 90.0 (83.0–94.0) | 78.0 (73.0–100.0) |
| Completed trials | Pre ITI | 86.0 (78.3–105.3) | 87.8 (85.3–118.3) | 96.7 (95.0–99.0) |
|  | Extended ITI | 62.0* (53.0–82.0) | 83.0* (77.0–90.0) | 73.0* (67.0–97.0) |
| P1% | Pre ITI | 58.8 (28.8–68.3) | 31.5 (20.5–39.3) | 76.2 (62.3–86.2) |
|  | Extended ITI | 47.1 (38.7–58.7) | 31.1 (19.5–37.1) | 82.1 (63.7–84.3) |
| P2% | Pre ITI | 20.1 (11.7–28.4) | 53.6 (39.0–55.9) | 7.2 (6.1–20.2) |
|  | Extended ITI | 26.5 (6.5–43.4) | 61.2* (46.7–68.8) | 6.2 (4.6–8.0) |
| P3% | Pre ITI | 9.7 (5.1–18.1) | 12.1 (7.5–14.3) | 4.6 (3.8–8.5) |
|  | Extended ITI | 17.1 (13.2–24.2) | 6.0* (2.2–14.3) | 6.5 (2.9–10.7) |
| P4% | Pre ITI | 13.4 (7.0–16.2) | 6.8 (0.9–9.8) | 8.3 (7.6–9.8) |
|  | Extended ITI | 6.5 (1.6–13.2) | 1.3 (1.1–4.8) | 4.6 (1.8–5.9) |

Results from the 7 s extended ITI on rGT behavior in rats with risky, strategic and safe strategies. Data are presented as median and quartile range. * < 0.05, ** < 0.01 comparing pre and extended ITI within the respective groups (*post hoc* Wilcoxon matched pairs test). Abbreviations: ITI, inter-trial interval; PR, premature response; PRdP, perseverative responses during punishment; PRdR, perseverative responses during reward.

**Table S4.** Correlation between choices in rGT1, rGT2 and rGT3.

|  | **All rats**  (n = 40) | | | | **Safe**  (n = 9–10) | **Strategic**  (n = 10) | **Risky**  (n = 7) | |
| --- | --- | --- | --- | --- | --- | --- | --- | --- |
| **rGT1 vs rGT 2** | P1% | P2% | P3% | P4% | P1% | P2% | P3% | P4% |
| P1% | 0.83* |  |  |  | 0.33 |  |  |  |
| P2% |  | 0.85* |  |  |  | -0.55 |  |  |
| P3% |  |  | 0.55* |  |  |  | 0.86* |  |
| P4% |  |  |  | 0.62* |  |  |  | 0.85* |
|  |  |  |  |  |  |  |  |  |
| **rGT2 vs rGT 3** |  | |  |  |  |  |  |  |
| P1% | 0.83* |  |  |  | 0.68* |  |  |  |
| P2% |  | 0.83* |  |  |  | 0.64* |  |  |
| P3% |  |  | 0.64* |  |  |  | 1.0 |  |
| P4% |  |  |  | 0.74* |  |  |  | 0.93* |
|  |  |  |  |  |  |  |  |  |
| **rGT1 vs rGT3** |  | |  |  |  |  |  |  |
| P1% | 0.80* |  |  |  | 0.62 |  |  |  |
| P2% |  | 0.87* |  |  |  | 0.14 |  |  |
| P3% |  |  | 0.44* |  |  |  | 0.86* |  |
| P4% |  |  |  | 0.65* |  |  |  | 0.79* |

Spearman rank order correlation coefficients (ρ) between rGT1 and rGT2, rGT2 and rGT3 as well as rGT1 and rGT3. Results are presented for all rats, as well as the characterizing choice for each rGT group (P1 for safe, P2 for strategic and P3 and P4 for risky). * < 0.05.

**Table S5.** Results from the three copulatory behavior tests in all rats irrespectively of rGT group.

| **Variable** | **n** | **Test 1** | **n** | **Test 2** | **n** | **Test 3** |
| --- | --- | --- | --- | --- | --- | --- |
| Latency mount* | 33 | 210.0 (50.0–360.0) | 37 | 35.0^11^ (20.0–125.0) | 36 | 17.5^111,2^ (7.5–42.5) |
| Latency intromission* | 36 | 142.5 (47.5–307.5) | 37 | 15.0^111^ (10.0–75.0) | 40 | 10.0^111^ (10.0–27.5) |
| Latency ejaculation* | 32 | 577.5 (420.0–750.0) | 36 | 445.0 (317.5–632.5) | 39 | 300.0^11,2^ (185.0–450.0) |
| Frequency mount* | 40 | 6.0 (3.0–10.5) | 40 | 11.5^111^ (8.0–17.0) | 40 | 11.0^111^ (6.0–16.0) |
| Frequency intromission* | 40 | 9.0 (6.0–12.0) | 40 | 11.5^11^ (9.0–15.0) | 40 | 12.0^111^ (10.5–14.5) |
| Frequency ejaculation* | 40 | 2.0 (0.0–2.0) | 40 | 2.0^11^ (1.0–3.0) | 39 | 3.0^111,2^ (2.0–3.0) |
| Post-ejaculatory interval 1 | 25 | 295.0 (270.0–305.0) | 32 | 277.5 (222.5–335.0) | 36 | 290.0 (262.5–315.0) |
| Post-ejaculatory interval 2 | 2 | 317.5 (290.0–345.0) | 18 | 275.0 (250.0–315.0) | 24 | 301.0 (285.0–337.5) |
| Post-ejaculatory interval 3 | 0 | 0.0 (0.0–0.0) | 2 | 307.5 (290.0–325.0) | 1 | 295.0 (295.0–295.0) |
| Mounts + intromissions* | 40 | 14.5 (10.0–20.0) | 40 | 23.5^111^ (18.0–30.5) | 40 | 24.0^111^ (18.0–30.0) |
| Intromission ratio | 36 | 0.6 (0.4–0.7) | 37 | 0.5 (0.4–0.6) | 40 | 0.5 (0.4–0.6) |
| Time from first intromission to ejaculation | 29 | 460.0 (325.0–555.0) | 36 | 417.5 (297.5–557.5) | 39 | 260.0^11,2^ (175.0–415.0) |
| Mounts + intromissions/ejaculation | 29 | 10.5 (8.0–13.0) | 36 | 12.5 (8.4–17.3) | 39 | 9.3 (7.3–13.5) |
| Inter-intromission rate | 29 | 50.0 (37.8–80.5) | 36 | 36.4^11^ (21.1–68.9) | 39 | 20.4^11^ (15.6–43.9) |
| Copulatory rate | 29 | 0.023 (0.019–0.034) | 36 | 0.034^111^ (0.028–0.045) | 39 | 0.040^111,2^ (0.028–0.057) |

Results from the three copulatory tests for all rats (n=40) irrespectively of rGT group. Data are presented as median and quartile range. Parameters indicated with * was included in the nparLD statistics, followed by Wilcoxon matched pairs tests were appropriate. The remaining parameters were analyzed with Friedman ANOVA followed by Wilcoxon matched pairs tests were appropriate. ^1^ < 0.05, ^11^ < 0.01, ^111^ < 0.001 compared with test 1, ^2^ < 0.05 compared with test 2.

**Table S6.** Average weekly (A) water and (B) total fluid intake during the 7 weeks of voluntary alcohol intake in rats with risky, strategic and safe rGT strategies.

| **Parameters** | **Risky (n = 7)** | | | | **Strategic (n = 10)** | | | | **Safe (n = 9)** | | | |
| --- | --- | --- | --- | --- | --- | --- | --- | --- | --- | --- | --- | --- |
|  | **Median** | **min – max** | | | **Median** | **min – max** | | | **Median** | **min – max** | | |
| **A.** Water intake (g/kg) |  |  |  |  |  |  |  |  |  |  |  |  |
| Week 1 | 45.1 | 39.2 | – | 53.4 | 43.8 | 35.4 | – | 47.3 | 45.1 | 40.9 | – | 54.4 |
| Week 2 | 44.6 | 30.8 | – | 66.6 | 49.7 | 43.4 | – | 56.6 | 48.1 | 32.0 | – | 57.5 |
| Week 3 | 42.7 | 30.3 | – | 62.0 | 48.1 | 25.9 | – | 60.4 | 45.4 | 33.1 | – | 58.0 |
| Week 4 | 43.4 | 29.2 | – | 56.0 | 46.4 | 32.9 | – | 50.8 | 43.9 | 39.3 | – | 57.4 |
| Week 5 | 49.2 | 29.5 | – | 58.4 | 43.5 | 37.9 | – | 57.9 | 46.6 | 40.3 | – | 57.6 |
| Week 6 | 37.8 | 29.4 | – | 43.8 | 45.4 | 38.8 | – | 59.6 | 44.5 | 39.7 | – | 51.9 |
| Week 7 | 43.1 | 31.5 | – | 45.4 | 37.8 | 33.3 | – | 50.6 | 42.5 | 35.6 | – | 50.0 |
| **B.** Total fluid intake (g/kg) | |  |  |  |  |  |  |  |  |  |  |  |
| Week 1 | 51.1 | 46.9 | – | 56.5 | 48.6 | 38.9 | – | 52.1 | 50.2 | 47.8 | – | 57.7 |
| Week 2 | 50.9 | 43.1 | – | 71.0 | 55.2 | 50.5 | – | 59.5 | 53.4 | 36.3 | – | 62.6 |
| Week 3 | 51.6 | 43.2 | – | 67.7 | 54.1 | 35.2 | – | 66.5 | 50.3 | 42.0 | – | 66.8 |
| Week 4 | 55.9 | 44.2 | – | 63.2 | 52.6 | 39.1 | – | 58.4 | 53.2 | 44.5 | – | 63.3 |
| Week 5 | 58.3 | 47.6 | – | 64.5 | 56.0 | 46.4 | – | 65.3 | 56.7 | 48.8 | – | 68.2 |
| Week 6 | 49.6 | 42.1 | – | 58.1 | 54.5 | 46.9 | – | 73.2 | 51.3 | 44.5 | – | 57.8 |
| Week 7 | 50.4 | 40.1 | – | 54.1 | 459 | 37.8 | – | 57.1 | 47.1 | 45.4 | – | 55.2 |

Intake measures during the 7 weeks of voluntary alcohol intake in rats with risky, strategic and safe rGT strategies. Data are presented as median and min – max. No significant differences between the groups were found.

**Table S7.** rGT results prior to (pre; mean of the 3 days prior to treatment) and during the naltrexone treatment in all rats irrespectively of rGT strategy, as well as in rats with risky, strategic and safe strategies.

| Parameter | Group | Pre | Saline | | | 0.3 mg/kg | | | 3.0 mg/kg | | |
| --- | --- | --- | --- | --- | --- | --- | --- | --- | --- | --- | --- |
|  |  |  | 30 min | 24 h | 48 h | 30 min | 24 h | 48 h | 30 min | 24 h | 48 h |
| Omissions | All | 20.6  (14.7–27.5) | 15.0***  (10.5–21.5) | 21.0  (15.0–23.0) | 18.0  (13.0–27.0) | 28.0***  (22.0–36.0) | 22.0  (16.5–27.0) | 20.0  (13.5–28.0) | 32.0***  (24.5–38.0) | 22.0  (18.0–28.0) | 21.0  (12.0–28.0) |
|  | Risky | 15.7  (10.3–25.0) | 11.0  (5.0–18.0) | 20.0  (9.0–21.0) | 15.0  (8.0–18.0) | 25.5  (14.0–34.0) | 17.0  (9.0–23.0) | 16.0  (12.0–19.0) | 30.5  (27.0–36.0) | 18.5  (9.0–22.0) | 10.5  (8.0–26.0) |
|  | Strategic | 24.2  (20.7–31.3) | 19.0  (14.0–27.0) | 22.5  (18.0–29.0) | 20.0  (15.0–28.0) | 34.5  (27.0–41.0) | 25.0  (19.0–27.0) | 23.0  (16.0–31.0) | 33.0  (24.0–36.0) | 24.0  (19.0–33.0) | 23.0  (16.0–27.0) |
|  | Safe | 25.0  (16.3–32.0) | 16.0  (11.0–26.0) | 21.0  (17.0–28.0) | 16.0  (14.0–30.0) | 24.0  (22.0–41.0) | 24.0  (20.0–29.0) | 23.0  (12.0–28.0) | 43.0  (31.0–55.0) | 24.0  (16.0–28.0) | 28.0  (18.0–31.0) |
| PR | All | 4.0  (1.6–5.7) | 8.5***  (6.0–14.0) | 2.0  (1.0–6.0) | 2.0  (1.0–6.0) | 2.5  (1.0–4.5) | 2.0**  (0.0–4.5) | 2.5  (1.5–6.0) | 1.0***  (0.0–3.0) | 2.0***  (1.0–4.0) | 2.0  (1.0–4.0) |
|  | Risky | 5.0  (3.3–6.3) | 7.5  (7.0–11.0) | 3.0  (1.0–6.0) | 2.0  (2.0–6.0) | 3.0  (2.0–4.0) | 2.5  (1.0–7.0) | 3.5  (1.0–7.0) | 1.0  (0.0–3.0) | 3.0  (1.0–4.0) | 3.0  (1.0–6.0) |
|  | Strategic | 3.3  (1.3–6.0) | 9.5  (3.0–14.0) | 2.5  (1.0–7.0) | 2.5  (1.0–7.0) | 2.5  (1.0–5.0) | 2.5  (0.0–5.0) | 3.0  (2.0–7.0) | 1.5  (0.0–6.0) | 2.0  (0.0–4.0) | 3.0  (1.0–4.0) |
|  | Safe | 4.7  (2.3–5.7) | 15.0  (2.0–23.0) | 2.0  (0.0–6.0) | 3.0  (0.0–8.0) | 2.0  (0.0–5.0) | 1.0  (0.0–3.0) | 2.0  (0.0–5.0) | 1.0  (0.0–3.0) | 1.0  (0.0–2.0) | 2.0  (0.0–11.0) |
| PRdP | All | 13.0  (9.7–18.3) | 24.0***  (15.5–31.5) | 12.0  (9.0–19.0) | 15.0  (9.0–21.0) | 14.0  (9.0–23.0) | 13.0  (8.5–17.0) | 14.0  (11.0–21.0) | 11.0**  (6.5–15.5) | 12.0**  (7.0–17.0) | 14.0  (8.0–20.0) |
|  | Risky | 18.2  (14.7–23.3) | 30.0**  (27.0–34.0) | 21.0  (12.0–36.0) | 24.0  (16.0–26.0) | 13.5  (6.0–15.0) | 16.5  (13.0–18.0) | 14.5  (8.0–21.0) | 13.0*  (11.0–16.0) | 16.5*  (13.0–19.0) | 16.5  (13.0–24.0) |
|  | Strategic | 11.4  (9.0–14.0) | 18.5*  (12.0–29.0) | 9.0  (8.0–14.0) | 9.5  (7.0–16.0) | 10.5  (6.0–17.0) | 8.5  (7.0–19.0) | 13.0  (10.0–19.0) | 7.5  (4.0–10.0) | 8.0  (6.0–11.0) | 14.0  (9.0–18.0) |
|  | Safe | 12.7  (9.7–16.7) | 22.0*  (15.0–43.0) | 13.0  (9.0–19.0) | 14.0  (7.0–18.0) | 17.0  (13.0–23.0) | 13.0  (9.0–15.0) | 14.0  (13.0–21.0) | 13.0  (9.0–17.0) | 14.0  (7.0–20.0) | 10.0  (6.0–17.0) |
| PRdR | All | 1.0  (0.3–1.5) | 2.0  (0.0–5.0) | 0.0  (0.0–1.0) | 0.0  (0.0–1.0) | 2.0  (0.0–4.0) | 0.0  (0.0–2.0) | 0.0  (0.0–1.0) | 0.0  (0.0–2.0) | 0.0  (0.0–2.0) | 0.0  (0.0–2.0) |
|  | Risky | 1.2  (0.3–2.0) | 3.5  (2.0–6.0) | 0.0  (0.0–1.0) | 0.0  (0.0–1.0) | 2.0  (0.0–3.0) | 0.5  (0.0–2.0) | 0.0  (0.0–1.0) | 0.5  (0.0–2.0) | 0.0  (0.0–2.0) | 0.0  (0.0–2.0) |
|  | Strategic | 0.5  (0.3–0.7) | 0.0  (0.0–2.0) | 0.5  (0.0–1.0) | 0.0  (0.0–0.0) | 0.0  (0.0–2.0) | 0.0  (0.0–1.0) | 0.0  (0.0–0.0) | 0.0  (0.0–0.0) | 0.0  (0.0–2.0) | 0.0  (0.0–1.0) |
|  | Safe | 1.0  (0.5–1.3) | 4.0  (0.0–12.0) | 2.0  (0.0–3.0) | 1.0  (0.0–11.0) | 4.0  (1.0–9.0) | 0.0  (0.0–4.0) | 1.0  (0.0–5.0) | 2.0  (0.0–4.0) | 0.0  (0.0–0.0) | 1.0  (1.0–3.0) |
| Head entries | All | 5.2  (2.0–12.2) | 12.5***  (4.5–27.0) | 6.0  (3.0–15.0) | 5.0  (1.0–12.0) | 4.5  (1.0–11.5) | 5.0  (1.0–9.5) | 4.5  (1.0–12.0) | 3.0**  (1.0–6.0) | 5.0  (1.0–11.0) | 6.0  (2.0–13.0) |
|  | Risky | 13.8  (9.3–22.0) | 26.0  (14.0–45.0) | 15.0  (8.0–26.0) | 16.0  (6.0–31.0) | 4.5*  (1.0–18.0) | 10.0  (5.0–25.0) | 7.0  (4.0–17.0) | 4.0**  (2.0–14.0) | 11.5*  (3.0–14.0) | 17.0  (2.0–26.0) |
|  | Strategic | 3.2  (2.0–5.3) | 10.5*  (7.0–22.0) | 4.5  (2.0–7.0) | 1.0  (0.0–5.0) | 3.5  (1.0–6.0) | 1.5  (0.0–10.0) | 1.5  (0.0–8.0) | 1.0  (0.0–4.0) | 3.0  (0.0–4.0) | 5.0  (0.0–10.0) |
|  | Safe | 4.3  (1.0–7.3) | 5.0*  (4.0–15.0) | 3.0  (2.0–8.0) | 2.0  (1.0–5.0) | 3.0  (0.0–9.0) | 4.0  (0.0–6.0) | 2.0  (0.0–13.0) | 3.0  (0.0–7.0) | 5.0  (1.0–7.0) | 4.0  (0.0–5.0) |
| Total trials | All | 90.3  (77.3–100.5) | 108.0***  (97.0–117.5) | 88.0  (80.0–103.0) | 84.0  (74.0–100.0) | 82.0*  (68.5–94.0) | 89.0  (73.0–98.0) | 90.0  (79.0–101.5) | 73.5***  (52.0–82.0) | 83.0**  (65.0–98.0) | 89.0  (78.0–98.0) |
|  | Risky | 88.3  (71.7–100.0) | 100.5**  (91.0–106.0) | 93.0  (74.0–101.0) | 81.0  (72.0–93.0) | 71.5*  (68.0–78.0) | 84.5  (58.0–93.0) | 85.0  (70.0–96.0) | 71.5*  (48.0–87.0) | 78.5**  (52.0–93.0) | 85.5  (69.0–100.0) |
|  | Strategic | 82.1  (77.3–97.3) | 103.5*  (93.0–116.0) | 86.5  (78.0–103.0) | 84.5  (75.0–98.0) | 77.0  (58.0–98.0) | 86.0  (74.0–95.0) | 91.5  (78.0–106.0) | 68.5**  (51.0–82.0) | 65.0*  (60.0–85.0) | 88.0  (84.0–92.0) |
|  | Safe | 100.3  (80.0–109.3) | 117.0*  (108.0–132.0) | 94.0  (87.0–111.0) | 95.0  (70.0–117.0) | 97.0  (83.0–105.0) | 98.0  (88.0–107.0) | 102.0  (85.0–107.0) | 85.0  (70.0–94.0) | 86.0  (73.0–111.0) | 97.0  (85.0–103.0) |
| Completed trials | All | 61.2  (48.7–74.0) | 81.0***  (69.0–92.5) | 65.0  (56.0–80.0) | 62.0  (49.0–74.0) | 47.0***  (40.0–57.5) | 63.5  (49.5–76.5) | 65.0  (51.0–82.5) | 33.0***  (21.5–41.5) | 55.0**  (43.0–73.0) | 63.0  (52.0–71.0) |
|  | Risky | 60.5  (48.3–69.7) | 79.5  (67.0–92.0) | 62.0  (59.0–80.0) | 53.0  (46.0–69.0) | 45.0  (33.0–48.0) | 62.5  (38.0–76.0) | 62.0  (44.0–73.0) | 38.0  (17.0–42.0) | 52.5  (36.0–64.0) | 64.5  (43.0–75.0) |
|  | Strategic | 55.2  (43.0–60.7) | 73.5  (66.0–81.0) | 64.5  (55.0–68.0) | 61.0  (49.0–74.0) | 39.0  (27.0–57.0) | 58.0  (50.0–68.0) | 58.5  (51.0–83.0) | 21.0  (17.0–41.0) | 40.0  (36.0–53.0) | 59.0  (57.0–63.0) |
|  | Safe | 68.7  (48.0–79.3) | 86.0  (81.0–93.0) | 71.0  (58.0–87.0) | 62.0  (43.0–93.0) | 64.0  (49.0–70.0) | 74.0  (64.0–83.0) | 77.0  (57.0–87.0) | 33.0  (30.0–40.0) | 59.0  (48.0–80.0) | 71.0  (57.0–75.0) |
| P1% | All | 27.5  (14.0–38.6) | 30.0  (13.6–41.2) | 24.1  (14.7–39.2) | 20.0  (8.3–35.9) | 31.9**  (16.7–50.4) | 21.6  (10.6–35.0) | 19.0**  (11.0–27.6) | 27.1  (11.3–48.7) | 22.2***  (8.2–34.4) | 18.6***  (5.7–34.0) |
|  | Risky | 30.0  (7.0–32.2) | 22.6  (5.4–33.7) | 17.5  (10.0–22.5) | 18.9  (7.6–23.9) | 27.5  (14.6–34.8) | 14.4  (6.3–23.9) | 12.6  (6.1–19.4) | 22.2  (12.5–30.8) | 17.4  (6.2–25.0) | 14.4*  (5.7–30.3) |
|  | Strategic | 10.9  (4.6–15.2) | 8.9  (2.5–21.2) | 10.9  (5.7–20.5) | 8.8  (5.4–15.6) | 12.4  (7.3–27.8) | 7.2  (2.9–14.8) | 6.9  (3.5–13.2) | 6.9  (0.0–10.0) | 3.6*  (0.0–10.5) | 4.0  (1.7–17.1) |
|  | Safe | 47.2  (39.5–60.3) | 56.2  (53.9–71.0) | 52.3  (46.6–58.1) | 48.8  (45.0–54.7) | 54.9  (49.0–72.3) | 45.2  (41.9–68.8) | 34.5*  (27.6–57.9) | 66.7  (53.7–77.5) | 47.9  (30.6–56.8) | 44.0  (34.0–61.3) |
| P2% | All | 62.1  (46.4–77.2) | 58.4*  (39.7–71.3) | 67.9  (44.2–75.8) | 63.0  (47.3–80.5) | 46.4***  (38.6–68.1) | 66.2  (48.3–76.4) | 70.5*  (58.0–79.9) | 50.4*  (34.5–70.6) | 61.3**  (47.9–84.2) | 64.3**  (43.6–82.5) |
|  | Risky | 46.4  (31.4–60.9) | 48.7  (33.3–63.3) | 64.4  (40.3–71.9) | 55.1  (41.5–59.5) | 40.3  (32.3–51.0) | 51.7  (36.8–65.8) | 60.3  (32.3–71.2) | 39.8  (33.3–50.0) | 47.0  (37.8–54.7) | 50.5  (28.0–62.1) |
|  | Strategic | 86.9  (78.9–90.5) | 83.9*  (71.7–93.9) | 78.8*  (73.0–92.9) | 86.5  (78.6–93.8) | 80.2*  (62.1–87.5) | 86.5  (77.8–96.8) | 87.7  (84.3–95.3) | 86.7  (72.7–94.7) | 94.5*  (84.2–100.0) | 95.6  (75.7–96.6) |
|  | Safe | 49.1  (38.3–56.7) | 32.5*  (28.0–41.6) | 44.2  (41.9–53.4) | 47.3  (40.0–52.5) | 38.8*  (23.4–43.8) | 48.8  (25.0–58.1) | 60.9  (35.1–69.0) | 33.3*  (20.0–42.6) | 49.2  (38.6–61.3) | 49.3  (38.7–63.4) |
| P3% | All | 2.5  (1.1–4.9) | 3.0  (1.2–8.0) | 2.0  (0.0–4.5) | 1.4  (0.0–4.7) | 2.7  (0.0–7.4) | 2.2  (0.0–5.3) | 1.8  (0.0–5.9) | 1.8  (0.0–6.4) | 2.2  (0.0–6.8) | 1.7  (0.0–5.6) |
|  | Risky | 8.4  (1.1–25.3) | 4.7  (1.2–23.9) | 2.5  (0.0–11.3) | 1.7  (0.0–17.4) | 5.0  (0.0–15.7) | 4.3  (0.0–21.2) | 3.8  (0.0–9.1) | 0.9  (0.0–26.8) | 5.9  (0.0–20.0) | 3.8  (0.0–32.2) |
|  | Strategic | 2.2  (0.0–2.7) | 3.0  (2.5–7.7) | 1.5  (0.0–4.5) | 0.0  (0.0–1.1) | 0.0  (0.0–3.8) | 1.0  (0.0–3.2) | 1.7  (0.0–1.9) | 0.9  (0.0–4.5) | 0.0  (0.0–1.8) | 1.6  (0.0–1.7) |
|  | Safe | 2.9  (1.9–4.6) | 3.9  (1.1–4.9) | 2.8  (0.0–3.5) | 0.0  (0.0–3.2) | 4.3  (1.6–6.1) | 2.7  (1.2–6.0) | 2.6  (1.1–4.6) | 1.9  (0.0–3.4) | 4.1  (2.1–5.4) | 1.8  (0.0–5.3) |
| P4% | All | 1.5  (0.2–4.4) | 1.5  (0.0–7.0) | 0.0  (0.0–4.8) | 1.3  (0.0–9.4) | 0.8  (0.0–9.1) | 0.6  (0.0–5.9) | 1.3  (0.0–6.4) | 2.1  (0.0–11.1) | 0.0  (0.0–3.5) | 0.0  (0.0–3.8) |
|  | Risky | 12.0  (0.7–27.5) | 9.1  (1.3–26.7) | 15.0  (0.0–35.6) | 16.7  (0.0–30.0) | 7.6  (0.0–30.6) | 7.8  (0.0–39.1) | 11.1  (1.4–31.3) | 10.8  (1.8–37.5) | 13.1  (0.0–28.1) | 10.7  (0.0–40.8) |
|  | Strategic | 0.4  (0.0–1.6) | 0.6  (0.0–1.5) | 0.0  (0.0–1.6) | 0.0  (0.0–2.0) | 0.0  (0.0–3.8) | 0.0  (0.0–1.9) | 0.0  (0.0–0.0) | 0.0  (0.0–0.0) | 0.0  (0.0–0.0) | 0.0  (0.0–0.0) |
|  | Safe | 0.5  (0.0–1.4) | 0.0  (0.0–1.1) | 0.0  (0.0–0.0) | 0.0  (0.0–1.1) | 0.0  (0.0–0.0) | 0.0  (0.0–1.2) | 0.0  (0.0–2.6) | 0.0  (0.0–1.9) | 0.0  (0.0–2.1) | 0.0  (0.0–1.4) |

Results of naltrexone treatment on rGT parameters obtained in rGT3 in all rats tested (n = 40) as well as in rats with risky (n = 7), strategic (n = 10) and safe (n = 10) rGT strategies. Data are presented as median and quartile range. * < 0.05, ** < 0.01, *** < 0.001 compared to the corresponding pre value (*post hoc* Wilcoxon matched pairs test). Abbreviations: PR, premature responses; PRdP, perseverative responses during punishment; PRdR, perseverative responses during reward.
